# ATF3 reduces migration capacity by regulation of matrix metalloproteinases via NF*κ*B and STAT3 inhibition in glioblastoma

**DOI:** 10.1038/cddiscovery.2017.6

**Published:** 2017-02-27

**Authors:** Jessica Guenzle, Louisa J Wolf, Nicklas W C Garrelfs, Jonathan M Goeldner, Nadja Osterberg, Cora R Schindler, Joseph E Saavedra, Astrid Weyerbrock

**Affiliations:** 1 Department of Neurosurgery, Medical Center-University of Freiburg, Faculty of Medicine, Breisacher Strasse 64, Freiburg D-79106, Germany; 2 University of Freiburg, Faculty of Biology, Schaenzlestrasse 1, Freiburg D-79104, Germany; 3 Cancer and Inflammation Program, Center for Cancer Research, National Cancer Institute (NCI) at Frederick, Frederick, MD 21702, USA

## Abstract

Glioblastoma is associated with poor survival and a high recurrence rate in patients due to inevitable uncontrolled infiltrative tumor growth. The elucidation of the molecular mechanisms may offer opportunities to prevent relapses. In this study we investigated the role of the activating transcription factor 3 (ATF3) in migration of GBM cells *in vitro*. RNA microarray revealed that gene expression of ATF3 is induced by a variety of chemotherapeutics and experimental agents such as the nitric oxide donor JS-K (O2-(2,4-dinitrophenyl) 1-[(4-ethoxycarbonyl)piperazin-1-yl]diazen-1-ium-1,2-diolate). We found NF*κ*B and STAT3 to be downstream targets inhibited by overexpression of ATF3. We demonstrate that ATF3 is directly involved in the regulation of matrix metalloproteinase expression and activation. Overexpression of ATF3 therefore leads to a significantly reduced migration capacity and induction of tissue inhibitors of matrix metalloproteinases. Our study for the first time identifies ATF3 as a potential novel therapeutic target in glioblastoma.

## Introduction

Glioblastoma multiforme (GBM) is the most frequent and malignant brain tumor with few therapeutic options and a poor clinical outcome. The understanding of the mechanisms that drive tumor progression plays a central role in the current GBM research, while elevated invasive capacity and multifocal growth are of great importance from a clinical point of view. Understanding the molecular regulatory mechanisms is therefore fundamental for the development of novel anti-tumor therapies to achieve tumor elimination. The ability to degrade extracellular proteins is essential for any individual cell to interact properly with its immediate surroundings. For multicellular organisms it is a prerequisite for development and chemotaxis.^1^ In physiological conditions, the extracellular matrix (ECM) is involved in wound healing and organogenesis; in pathological conditions it is significantly involved in local tumor progression and metastasis in cancer.^2,3^ Migration and metastasis are complex interactions between cells and the ECM. There are various groups of proteolytic enzymes or proteases involved in matrix degradation, but the matrix metalloproteinase (MMP) group of enzymes is the most important in the context of tumor invasion and metastasis.^2,4^ The MMPs are zinc and calcium-dependent endopeptidases that modify signaling pathways and cell functions under physiological and pathological conditions.^5^ MMPs are involved in migration, inflammation, proliferation, apoptosis and differentiation and are therefore latently synthesized.^5,6^ The substrates of MMPs are specific proteins of the ECM like collagen, fibrin, integrins and receptors.^2,7^ Various studies demonstrated a correlation between expression of MMPs and the malignancy and invasive capacity of GBM.^8^ Certain MMPs such as MMP1 and MMP7 are associated with a poor prognosis in cancer.^5,9,10^ MMPs are secreted in the ECM as inactive zymogens interacting with tissue inhibitors of metalloproteinases (TIMP).^4^ TIMPs bind to the catalytic domain of MMPs and can inhibit the ‘cysteine-switch’, which disrupts the interaction between a cysteine in the prodomain and the Zn^2+^ ion in the active site. After the cleavage of the complex the prodomain of the MMP is released for activation.^11–13^ In general, each TIMP can form complexes with each MMP but there are preferential interactions with higher affinity between some of them.^14^ The 28 MMPs described in human are classified by the prodomain, amino acid sequence and substrate specificity.^2^ The AP-1 binding site has been considered to play an important role in the transcriptional activation of the MMP promoters. Activating transcription factor 3 (ATF3) is a stress-induced and adaptive-responsive gene that encodes a member of the ATF/CREB super family of transcription.^15^ The basal level of ATF3 is very low but can rapidly increase under stress. ATF3 is activated by forming a homodimer or heterodimers with ATFs or proteins of the CREB/CREM and AP-1 families, which contain consensus sequences.^16^ ATF3 functions are variable in a context-dependent manner, therefore research efforts to delineate its function are difficult. DNA damage, non-steroidal anti-inflammatory drugs, progesterone and even mere cellular stress can induce ATF3 expression and activation.^17,18^ Recent studies report that ATF3 has neuroprotective functions, for example, through downregulation of inflammatory cytokines or prevention of apoptosis.^16,17^ Overexpression of ATF3 in lung cancer cells leads to reduced metastasis, whereas knockdown of ATF3 results in a higher migration capacity with increased levels of actin filaments and correlates with a shorter overall survival in esophageal squamous cell carcinoma.^19,20^ Since ATF3 is known to be stress inducible, it is conceivable that it is regulated by factors involved in stress response and inflammation such as nitric oxide (NO). NO is a free radical with diverse functions in immunoreactions and neurotransmission.^21^ Endogenous NO is generated by eNOS and nNOS under physiological conditions in the brain and may act as a cytoprotective and proliferative element in neuronal cells.^22,23^ One explanation for this cytoprotection is the ability of NO to mediate cGMP generation and therefore the differentiation of precursor cells and prevention of apoptosis after stimulation which could be confirmed in myogenic cells.^24–26^ To stabilize the reactive and diffusible NO and to facilitate delivery of therapeutic NO doses, a prodrug was developed for *in vitro* and *in vivo* usage. The prodrug JS-K (O2-(2,4-dinitrophenyl) 1-[(4-ethoxycarbonyl)piperazin-1-yl]diazen-1-ium-1,2-diolate) is a diazeniumdiolate that releases NO after enzymatic metabolization by glutathione S-transferases (GSTs).^27^ In previous studies we could show the specific release of NO by JS-K in GST-overexpressing GBM cells affecting their proliferation activity and viability in a dose- and time-dependent manner.^28^ Furthermore, we could show that cells exposed to high concentrations of NO undergo mitotic catastrophe resulting in necrosis.^29^ Nitric oxide (NO) in low concentrations inhibits induction of cell death by *S*-nitrosylation of signaling molecules such as NF*κ*B, caspases and transcription factors.^30–32^ There is some evidence that NO released by NO donors at non-cytotoxic doses affects the migratory capacity of tumor cells.^33,34^

The main objective of this study was to investigate the molecular mechanisms of migration induced by low dose NO released from the diazeniumdiolate JS-K in glioma cells *in vitro*. ATF3 was found to be upregulated by NO in RNA microarray. In this study we provide evidence that overexpression of ATF3 correlates with expression and activation of MMPs and TIMPs *in vitro*. The reduced activation of MMPs leads to restricted migration capacity of established and primary GBM cells *in vitro*. In addition, we demonstrate a positive correlation between ATF3 and phosphorylation of STAT3 and the inhibition of nuclear translocation of NF*κ*B in glioblastoma cells. We show that the transcription factor ATF3 directly regulates gene expression of *nfκb*, *stat3* and *klf6* that are involved in tumor progression and invasion. Therefore, we propose that ATF3 could serve as prognostic marker for migration and metastatic disease in glioblastoma.

## Results

### Nitric oxide reduces migration capacity in a time- and dose-dependent fashion

**Figure 1:**
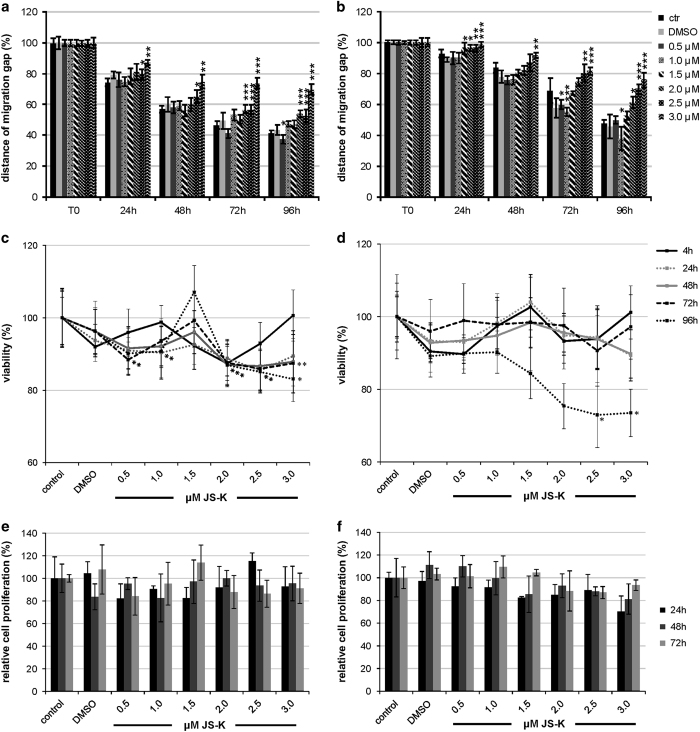
Inhibition of migration induced by 0.5–3.0 *μ*M JS-K in U87 (**a**) and primary IC cells (**b**). Migration capacity was measured by wound closure assay over 96 h. The initial width of the migration gap was 2 mm. JS-K treated cells and DMSO controls are normalized to T0 (start of experiment) set to 100% (±S.D. of three independent experiments). Asterisks indicate significance between treatment and controls. The non-toxic effect of low dose JS-K (0.5–3.0 *μ*M) and DMSO control on U87 (**c**) primary IC cells (**d**) after 48 h was determined by MTT assay. Viability of treated cells was plotted relative to the viability of untreated controls set to 100% (±S.D. of three independent experiments). BrdU incorporation assay exhibited no significant antiproliferative effect induced by JS-K (0.5–3.0 *μ*M) on U87 (**e**) and primary IC cells (**f**) over 72 h. Treatment was normalized to untreated controls (±S.D. of three independent experiments). Asterisks (*P<0.05, **P<0.01, ***P<0.001) indicate significance between JS-K and controls.

To investigate the influence of JS-K on migration capacity of glioma cells, we performed a wound closure assay over 96 h for U87 and primary IC cells (Figure 1). Starting at 24 h, the ability to close the migration gap is time-dependently reduced in both cells lines. In U87 cells, this significantly reduced effect can be observed at a concentration of 2 *μ*M JS-K (Figure 1a), whereas in primary IC cells it emerged at 1 *μ*M (Figure 1b). Up to 3 *μ*M JS-K progressively inhibited migration in a dose-dependent manner.

### Migration capacity is not influenced by reduction of viability and proliferation

One explanation for cells not closing a wound can be increased cell death elicited by the treatment or the inhibition of proliferation. To exclude these factors caused by JS-K we investigated cell viability by MTT assay over 96 h as well as the proliferation rate by BrdU incorporation assay (Figures 1c–f). Less than 20% of U87 cells were killed at the highest dose of 3 *μ*M after 96 h (Figure 1c). Viability of primary IC cells was not affected by JS-K up to 72 h after treatment (Figure 1d). At 96 h, the viability decreased to 68% when using 3 *μ*M JS-K. In contrast, no influence of JS-K on proliferation was detected at the tested doses at all (Figures 1e and f). At no time point either cell line showed a significant reduction or inhibition of proliferation.

### Microarray analysis reveals activating transcription factor 3 to be upregulated by NO

**Figure 2:**
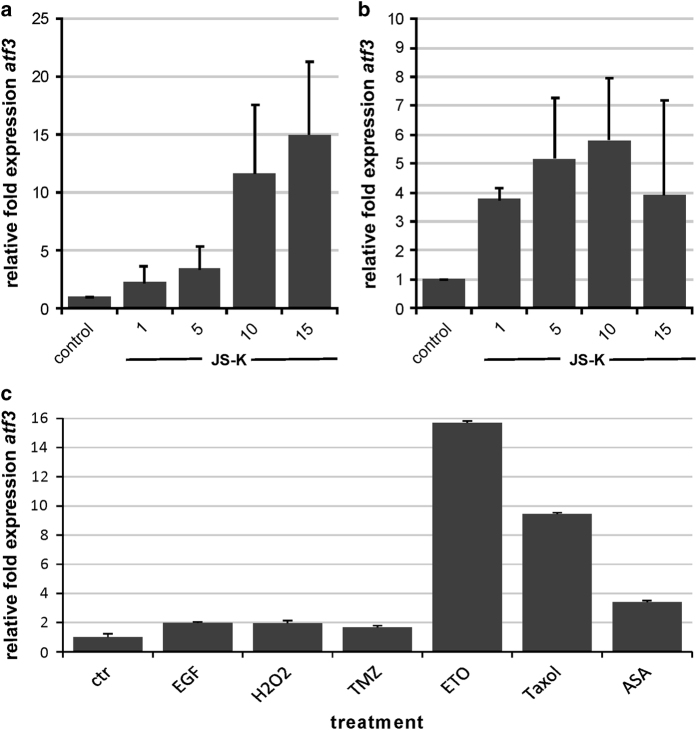
Dose-dependent relative gene expression of ATF3 in U87 (**a**) and primary IC cells (**b**) induced by JS-K up to 15 *μ*M for 48 h. Expression was determined by qRT-PCR and data show mean±S.D. of triplicate normalized to untreated controls. Furthermore, induction of ATF3 by chemotherapeutic agents was investigated by qRT-PCR. Cells were exposed to 100 ng EGF, 200 *μ*M H_2_O_2_, 50 *μ*M TMZ, 100 *μ*M Etoposide, 50 *μ*M Taxol and 10 mM ASA for 4 h. Expression was normalized to untreated controls set to 1.

To identify the gene targets of NO, we determined the temporal gene expression profile of U87 cells exposed to NO for 48 h using RNA microarray. We used U87 cells as a test cell line because this cell line is well characterized for many years as a standard for biochemical and genetic experiments in glioblastoma research. The data set was analyzed for upregulated and downregulated genes more than 2-fold in a statistically significant manner (*P*≤0.05). Approximately 10 genes were found to be upregulated (Table 1) and further analyzed by qRT-PCR to validate the array (data not shown). Activation transcription factor 3 (ATF3) was found to be upregulated 2.51-fold in the microarray. Primary IC cells and U87 cells revealed dose-dependent upregulation of expression in qRT-PCR after 48 h exposure to JS-K at concentrations up to 15 *μ*M (Figure 2). U87 cells showed a 15-fold expression compared to controls at the highest concentration of 15 *μ*M (Figure 2a). Primary IC cells had the highest peak of 5.8-fold expression when using 10 *μ*M JS-K (Figure 2b). ATF3 is known for its key role in suppression of metastasis and invasion. Therefore we chose migration for further investigation of the role of ATF3 in glioblastoma. To demonstrate the relevance of ATF3 we examined ATF3 RNA expression in response to treatment with various anticancer agents (Figure 2c). Epidermal growth factor (EGF) and hydrogen peroxide (H_2_O_2_) which is known to induce necrosis, did not induce the expression of ATF3 more than 2-fold. Temozolomide (TMZ), a common chemotherapeutic agent used in GBM therapy, did not even induce a twofold upregulation in contrast to Etoposide (ETO), also used for GBM therapy, leading to a 16-fold expression of ATF3. Paclitaxel (Taxol), a chemotherapeutic for a variety of tumors, showed a 9.5-fold expression and the anti-inflammatory acetylsalicylic acid (ASA) a 3.4-fold expression of ATF3. This highlights the considerable importance of research into ATF3 in glioblastoma.

### Overexpression of ATF3 reduces migration capacity and proliferation *in vitro*

**Figure 3:**
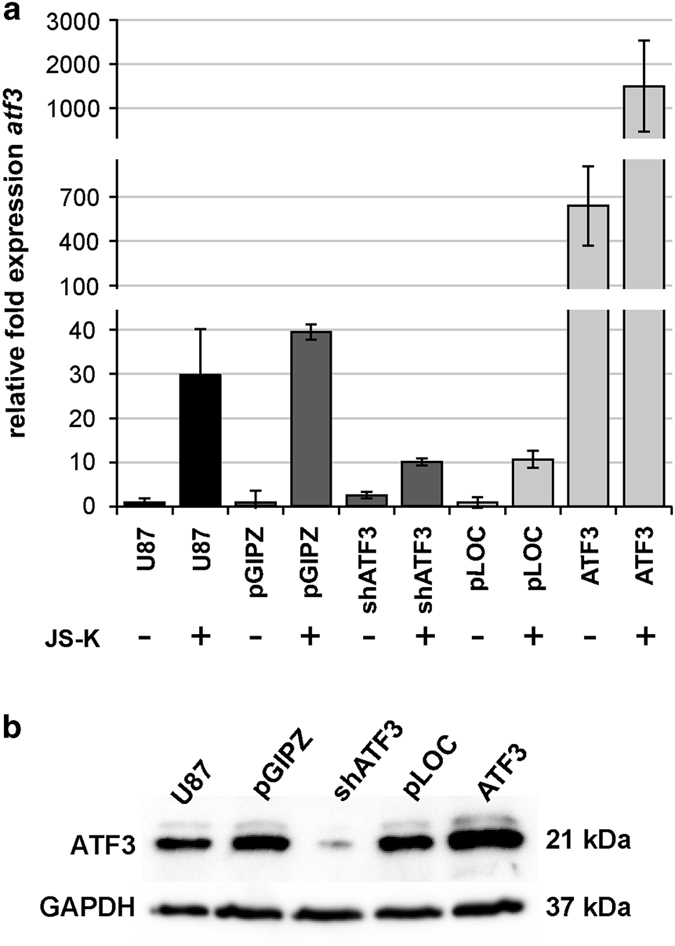
Efficiency of ATF3 knockdown and overexpression in U87 cells. U87 cells were stably infected with lentiviral particles containing shRNA for ATF3 or ATF3 and controls. (**a**) Target gene expression was measured by qRT-PCR and normalized to controls pGIPZ (shATF3) or to pLOC (ATF3) set to 1. In addition, cells were treated with 15 *μ*M JS-K for 48 h to induce ATF3 expression. ATF3 was inducible in uninfected U87 and in controls pGIPZ and pLOC. (**b**) The protein level of ATF3 was examined by western blot analysis in overexpressing and knockdown cells. GAPDH was used as loading control. Figures are representative for three independent experiments.

**Figure 4:**
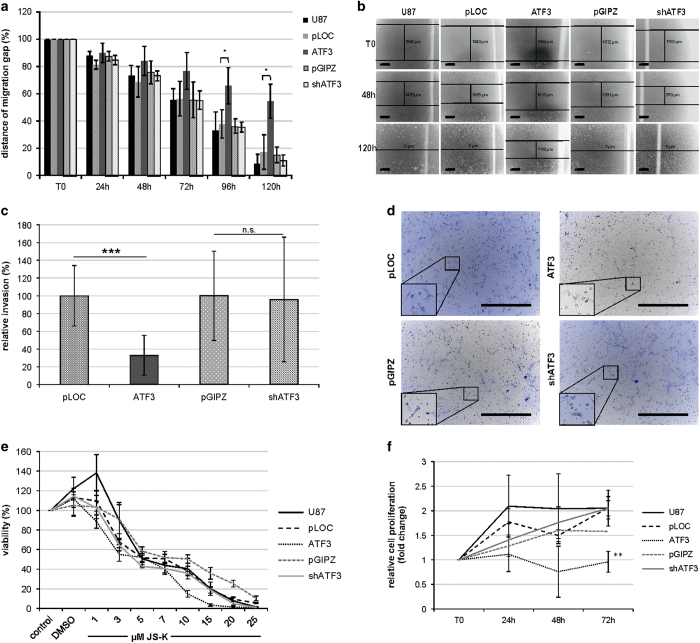
Migration and invasion of knockdown and overexpression of ATF3 was investigated by wound closure assay and Boyden Chamber assay. (**a**) shows the distance of the migration fronts over 120 h set to T0 (start of experiment, 100%). Representative images of the migrating cells exhibited the total width of the wound at T0, 48 and 120 h for untreated and plasmid controls (pLOC, pGIPZ), ATF3 and shATF3 (**b**). Scale bar indicates 500 *μ*m. Invasive capability of infected cells was quantified from Matrigel assays relative to controls set to 100% (±S.D. of three independent experiments) (**c**). Representative images of invading cells are shown in **d** for plasmid controls (pLOC, pGIPZ), ATF3 and shATF3. Scale bar indicates 500 *μ*m, frames show ×2.5 magnification. Response to JS-K (up to 25 *μ*M) by cells with knockdown and overexpression of ATF3 was measured by MTT assay (**e**). Viability of treated cells was plotted relative to viability of untreated controls set to 100% (±S.D. of three independent experiments). Relative cell proliferation of knockdown and overexpression was examined in BrdU incorporation assay over 72 h (**f**). Proliferation was normalized to T0 (start of experiment) set to 1. Asterisks (*P<0.05, **P<0.01, ***P<0.001) indicate significance to controls or T0.

Since we observed the overexpression of ATF3 in GBM cells induced by NO as well as different anticancer agents, we overexpressed and knocked down ATF3 stably by lentiviral infection in U87 cells. The efficacy of the knockdown (shATF3) and the overexpression (ATF3), done with their controls, pGIPZ and pLOC, are shown in Figures 3a and b. Knockdown, overexpression and treatment with JS-K are normalized to the particular control set to one. The controls still react to JS-K as the uninfected cells (U87). The knockdown of ATF3 led to a decreased ATF3-expression to 25% compared to control. The overexpression showed RNA levels of more than 650-fold compared to control and could be still increased by JS-K. To demonstrate the effect of ATF3 on migration of U87 cells we repeated the wound closure assay. Cells infected with pLOC, pGIPZ and shATF3 did not show any difference in migration capacity compared to uninfected U87 cells over the 120 h observation period (Figures 4a and b). However, ATF3-overexpressing cells migrated significantly slower into the gap (Figures 4a and b). While all cells not overexpressing ATF3 closed the gap within 120 h, the ATF3 overexpressing cells still exhibited a gap of about 1 mm, which accounts for 50% of the initial space (Figure 4b). To reveal an association between reduced migration and reduced viability of ATF3 overexpressing cells, we performed Matrigel invasion assay. Compared to controls, knockdown of ATF3 did not lead to altered invasive capability, whereas the overexpression of ATF3 decreased invasion of the cells significantly (*P*=0.00004) to 32% (Figures 4c and d). Since ATF3 is upregulated by NO, we expected a different behavior to JS-K in cell death induction, especially of shATF3. Cell lines showed no difference in viability when exposed to JS-K up to 25 *μ*M for 48 h in the MTT assay (Figure 4e). The relative cell proliferation measured by the BrdU incorporation assay indicated a significant reduction in ATF3 cells after 72 h (Figure 4f). Both controls and shATF3 showed the same proliferation rate compared to uninfected U87.

### Overexpression of ATF3 does not regulate mRNA expression of migration-related genes but prevents translocation of NF*κ*B and STAT3 phosphorylation

**Figure 5:**
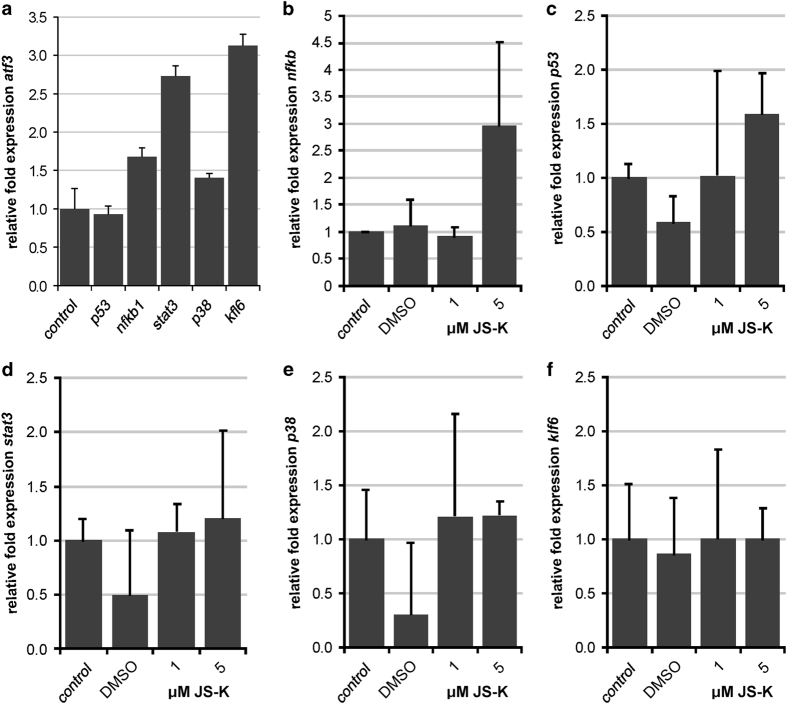
Relative gene expression of ATF3 overexpressing cells was determined by qRT-PCR. Expression of *p53*, *nfκb1*, *stat3*, *p38α* and *klf6* in ATF3 overexpressing U87 cells was normalized to controls of uninfected U87 cells (**a**). Treatment with 1–5 *μ*M JS-K for 48 h was performed in ATF3 overexpressing cells and pLOC controls (**b**–**f**). Relative gene expression of *nfκb1* (**b**), *p53* (**c**), *stat3* (**d**), *p38α* (**e**) and *klf6* (**f**) was investigated by qRT-PCR. Expression of pLOC was subtracted from expression of ATF3 overexpression to demonstrate the exclusive effect of ATF3 on target gene mRNA. Data were normalized to untreated controls±S.D. of triplicate.

**Figure 6:**
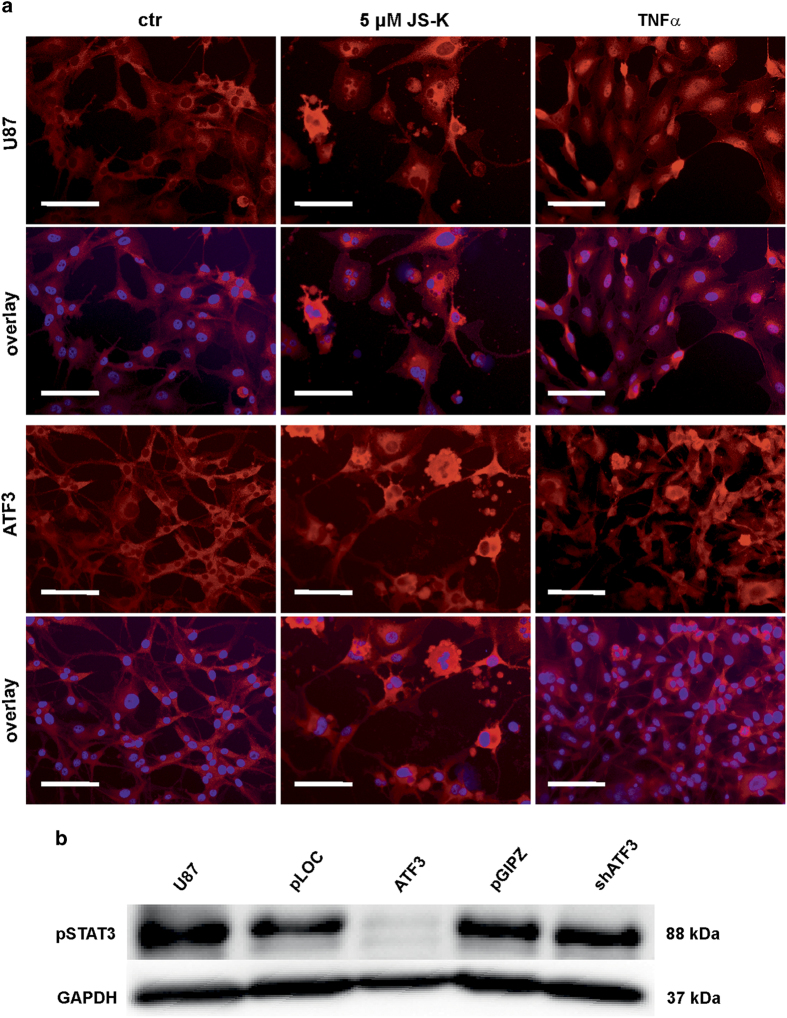
Representative images of nuclear translocation of NF*κ*B in uninfected U87 and ATF3 overexpressing U87 cells (**a**). Translocation was examined by immunocytochemistry for p65 (red) and DAPI (blue). Cells were treated with 5 *μ*M JS-K for 48 h or TNF*α* for 6 h and compared to untreated controls. Scale bar indicates 200 *μ*m. Phosphorylation of STAT3 was investigated by Western blot analysis for ATF3, shATF3 and controls (**b**). GAPDH was used as loading control. The figures shown are representative of three independent experiments.

The transcription factor ATF3 is known to regulate the expression of a number of genes. To investigate the relevance of ATF3 for migration we performed qRT-PCR for *p53*, *nfκb1*, *stat3*, *p38α* and *klf6*. Figure 5a shows the regulation of these genes in ATF3 overexpressing U87 compared to uninfected cells. S*tat3* and *klf6* were upregulated more than 2-fold in response to overexpression of ATF3 but not *p53*, *nfκb1* and *p38α.* However, even if *stat3 mRNA* expression was upregulated, Western blot revealed that STAT3 is no longer phosphorylated with ATF3 overexpression (Figure 6b). When further treated with up to 5 *μ*M JS-K, we observed a threefold upregulation of *nfκb1 mRNA* expression after 48 h (Figure 5b) but no difference in *p53* (Figure 5c), *stat3* (Figure 5d), *p38α* (Figure 5e) and *klf6* (Figure 5f). Since the NF*κ*B pathway plays an important role for migration and invasion of tumor cells, we further investigated the activation of NF*κ*B. Immunocytochemistry in cells treated with 5 *μ*M JS-K for 48 h showed no translocation of NF*κ*B (p65) into the nucleus caused by NO in either cell line, control or overexpressing ATF3 (Figure 6a). p65 could be detected in the cytoplasm with a much higher protein level in NO-treated cells compared to controls. Treatment with TNF*α* is well known to induce translocation of p65 and was used in this experiment as a positive control of translocation. Control cells translocate p65 after stimulation with TNF*α* for 6 h but not cells overexpressing ATF3 (Figure 6a). NF*κ*B (p65) remained in the cytoplasm and was barely detectable in the nuclei.

### Overexpression of ATF3 regulates protein level of matrix metalloproteinases and tissue inhibitors of metalloproteinases 3

**Figure 7:**
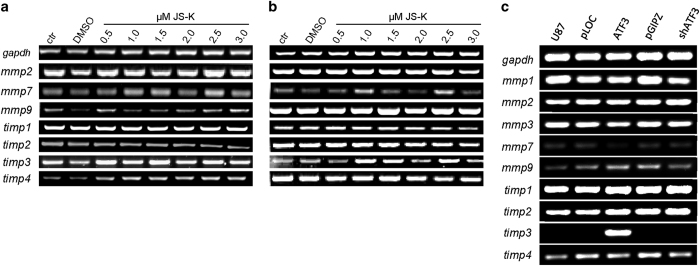
PCR of migration-related genes was performed to assess gene expression alterations underlying the changes in migration capacity. U87 cells (**a**) and primary IC cells (**b**) were treated with JS-K (0.5–3.0 *μ*M) for 48 h and tested for *mmp2*, *mmp7*, *mmp9* and *timp 1–4* expression. Cells exhibiting knockdown or overexpression of ATF3 were additionally tested for expression of *mmp1* and *mmp3*. GAPDH was used as loading control. Figures show representative images of triplicates.

Matrix metalloproteinases (MMPs) stimulate tumor metastasis by the degrading extracellular matrix. In this study we found no visible regulation of RNA expression in PCR caused by NO in MMP2, MMP7 and MMP9, nor in their inhibitors TIMP1 and TIMP3 in either U87 (Figure 7a) or primary IC cell lines (Figure 7b). TIMP2 was slightly reduced by increasing concentrations of JS-K in U87, whereas TIMP4 was dose-dependently enhanced by NO (Figure 7a). In U87 cells with ATF3 knockdown we did not observe any difference in expression of MMP1–3, MMP7 or MMP9 compared to pGIPZ or uninfected controls (Figure 7c). In contrast, cells overexpressing ATF3 exhibited no RNA expression of MMP7 compared to pLOC and uninfected cells. TIMP3 was significantly overexpressed in ATF3 overexpressing cells.

**Figure 8:**
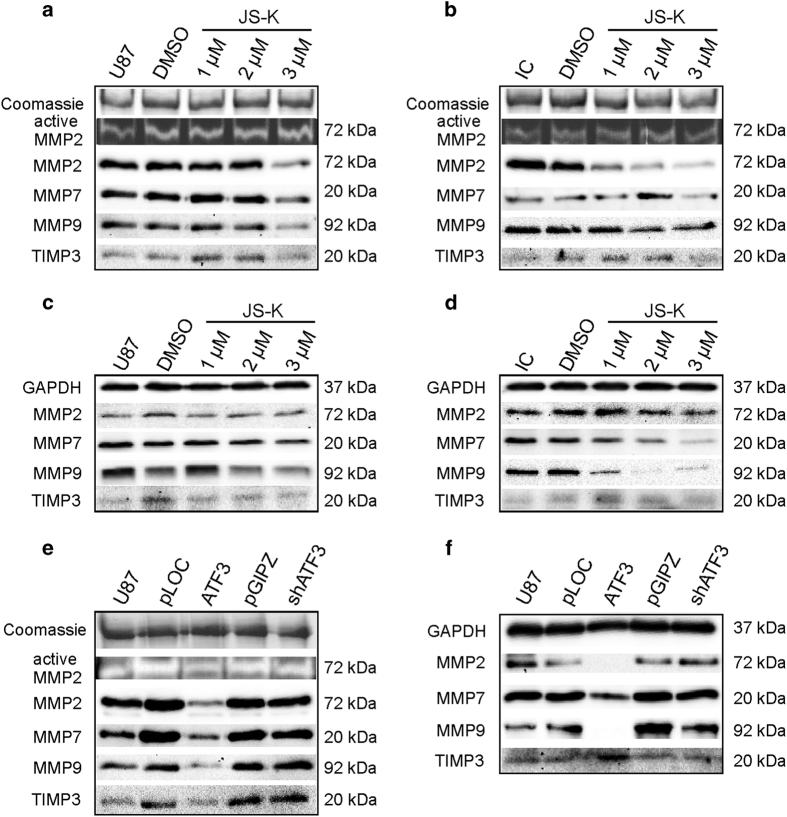
Zymography analysis for active MMP2 was performed with 4 *μ*g of total protein of the conditioned medium of U87 (**a**) and primary IC cells (**b**) after 48 h exposure to JS-K (1–3 *μ*M) on gelatin-containing PAGE. Western blot analysis for MMP2, MMP7, MMP9 and TIMP3 was performed for conditioned medium of U87 (**a**) and IC (**b**) cells with 4 *μ*g of total protein as well as of 20 *μ*g of total cell lysate of U87 (**c**) and IC cells (**d**) on SDS-PAGE. Conditioned media of knockdown and overexpression of ATF3 were examined for active MMP2 as well as the plasmid controls and uninfected U87 (**e**). 4 *μ*g of protein of the supernatant (**e**) and 20 *μ*g of cell lysates (**f**) were screened for MMP2, MMP7, MMP9 and TIMP3 by SDS-PAGE. Coomassie staining of the gels was used as a loading control for zymogram and for western blot of the supernatant, GAPDH was used as a loading control for cell lysates. Figures are representative for three independent experiments.

Since MMP2, MMP7 and MMP9 are important for the degradation of the tumor matrix, we investigated their protein levels by Western blot and the activity of MMPs by zymography (Figure 8). Western blot of the conditioned media obtained from U87 and primary IC cells showed a reduced protein level of MMP2, MMP7 and MMP9 with increasing concentrations of JS-K up to 3 *μ*M and an enhanced expression of TIMP3 at 1 and 2 *μ*M in U87 and primary IC cells (Figures 8a and b). The whole cell lysate of U87 cells exhibited an almost equal expression level of MMP2, MMP7 and TIMP3 but a dose-dependently decreasing level of MMP9 (Figure 8c). However, whole cell lysates of primary IC cells revealed decreasing protein levels of MMP2, MMP7 and MMP9 with low dose JS-K and no change in TIMP3 expression (Figure 8d). The protein level of MMP2, MMP7 and MMP9 in the supernatant of ATF3 overexpressing cells was considerably reduced compared to pLOC controls as well as the protein level of TIMP3 (Figure 8e). Western blot of the whole cell lysate of ATF3 knockdown and overexpression indicated a total deficiency of MMP2 and MMP9 in ATF3 overexpressing cells and a lower protein level of MMP7 (Figure 8f). In contrast, the inhibitor TIMP3 was overexpressed in ATF3 cells compared to controls and knockdown (Figure 8f). Zymography analysis of the conditioned media revealed no change in the activation of MMP2 in U87 exposed to NO (Figure 8a), whereas IC cells exhibitied less activation when treated with 3 *μ*M JS-K (Figure 8b). However, ATF3 overexpression reduced the level of active MMP2 in the supernatant of the cells compared to ATF3 knockdown and controls (Figure 8c). MMP7 and MMP9 could not be detected in gelatin or casein gels (data not shown).

## Discussion

Exploration of molecular mechanism in GBMs is of great importance for a full understanding of the biology of this highly malignant cancer and the identification of potential therapeutic targets. Recent research suggest that NO is a putative adjuvant in anti-tumor therapy but some mechanisms remain unclear so far.^35–37^ To obtain a new insight into the role of NO in glioblastoma biology and treatment, we investigated its role on gene targets and molecular mechanisms of cell migration. NO is known for its antiproliferative and cytotoxic effects in glioblastoma cells at high concentrations and its impact on migration, invasion and angiogenesis in various tumor cells at low doses.^28,29,38^ JS-K is an arylating agent designed to release nitric oxide upon reaction with glutathione in the presence of glutathione S-transferase, an enzyme overexpressed in cancer cells.^25,28,39^ Thus, JS-K application in GBM cells allows a cell type-specific intracellular NO release. The impact of high dose JS-K was described by our group as dose- and time-dependent concerning the inhibition of proliferation and induction of cell death.^28,29,40^ NO induces DNA double-strand breaks and post-translational modifications of proteins, such as *S*-nitrosylation of NF*κ*B resulting in activation of caspase-dependent and caspase-independent pathways.^31,32,38^ In previous studies we observerd the strongest cellular effects in glioblastoma cells after 48 h of JS-K treatment. In this study we found the transcription factor ATF3 to be upregulated by 15 *μ*M JS-K by RNA microarray. We provide evidence in established and primary cell lines that ATF3 is dose- and time-dependently regulated by NO in concentrations between 1 and 15 *μ*M. In cell culture, we could observe that the migration capacity was reduced under treatment with JS-K. While cell viability and proliferation are not affected by up to 3 *μ*M JS-K, cells migrated slower in a dose- and time-dependent fashion. Cell migration and invasion are mainly driven by MMPs *in vivo*.^6,9^ Recent research demonstrates that AP-1 protein complexes are involved in expression and activation of MMPs.^4,41,42^ Since ATF3 is known to form heterodimers with AP-1 proteins, we hypothesize that ATF3 is a direct regulator of MMP expression and activation. Cells secrete MMPs as zymogens into the ECM where they must be activated through enzymatic cleavage. By western blot we could demonstrate that NO induces protein expression and secretion of MMP2, 7 and 9 dose dependently in the supernatant of the cultured cells. This dose-dependent capacity indicates that NO is not activating a long signaling cascade but acts in a more direct way on the gene expression. The reduced expression of MMP2, 7 and 9 in cell lysates as well as in the supernatant of ATF3 overexpressing cells demonstrates the direct impact of the transcription factor on proteins involved in migration and invasion. Since RNA expression of MMPs is not affected by the dose-dependent regulation of NO and ATF3, we conclude that the principle of regulation of MMPs must occur on a post-translational level. The activation of MMPs can be investigated by zymography.^4^ In the conditioned media of cells exposed to NO we observed a dose-dependent reduction of activity of MMP2 in both cell lines, which implies a reduced cleavage of the ECM and therefore less migration and invasion capacity.^3^ Activation of MMP2 is significantly reduced when ATF3 is overexpressed but we could not observe any difference when ATF3 is knocked down. The efficiency of the ATF3 knockdown in U87 cells was at about 75%. qRT-PCR revealed that ATF3 is basically less expressed in established and primary cell lines. However, the remaining 25% of expressed ATF3 can still be sufficient to regulate invasive processes. Due to the same specific substrate of the gelatinases, we expected to induce activation of MMP2 as well as MMP9, but we were not able to detect active MMP9 in the gelatin gels under a variety of conditions. The activation of the matrilysin MMP7 can be monitored by PAGE containing casein.^5^ Both in the supernatant of cells exposed to NO and in ATF3 overexpressing cells only a weak but equal signal was detectable. We could not observe any difference induced by NO or ATF3 (data not shown). The zymogens are released from the cytoplasm of tumor cells where they are inhibited by TIMPs.^13,14^ The excessive cleavage of the ECM associated with an imbalance of the MMPs/TIMPs ratio has been correlated with the invasive potential of brain tumor cells.^7,43^ We could observe by PCR that TIMP3 is highly expressed in cells overexpressing ATF3 but not following exposure to JS-K up to 3 *μ*M. We conclude that the regulation of the inhibitors is not directly affected by NO and may need stronger promotor induction. However, we could detect that protein expression of TIMP3 also increased by NO in both the cell lysate and the supernatant of the cells. In ATF3 overexpressing cells, we could detect overexpressed TIMP3 in the lysate but not in the supernatant. This leads us to the assumption that TIMP3 is released from MMP binding during secretion from the cells when ATF3 is overexpressed. Migration and invasion are regulated by several signaling pathways. NF*κ*B1, KLF6, p53 and p38*α* are known to be involved in cell migration and invasion.^44–48^ Gene expression of *nfκb1* is not directly affected by ATF3 as observed by qRT-PCR. However, in cells overexpressing ATF3, the nuclear translocation of NF*κ*B could no longer be induced by TNF*α*. In contrast, NO induces *nfκb1* gene expression and protein expression of p65 even in ATF3 overexpressing cells but it is not translocated into the nuclei by NO. This indicates that the role of NO is not based on the same signaling pathways as ATF3. Marshall *et al.* and other groups found in lung carcinoma that NF*κ*B is nitrosylated and therefore inhibited by NO *in vitro*.^31,49^ In previous studies the *S*-nitrosylation of proteins induced by JS-K was well investigated and we conclude that nuclear translocation of NF*κ*B is inhibited by direct interaction with NO.^50,51^ The gene expression of tumor protein *p53* plays an important role in migration research. Yan *et al.* found that ATF3 activates p53 in colon carcinoma cells.^52^ In our study, *p53* is controlled neither by ATF3 nor by NO in low doses. Translocation of p53 into the nuclei is also not affected by ATF3 or NO (data not shown). Xu *et al.*^53^ found that KLF6 was upregulated by ATF3 in glomerular mesangial cells to induce apoptosis. Ahronian *et al.*^46^ identified KLF6 as a potent regulator of migration in hepatocellular carcinoma cells. Gene expression of *klf6* is significantly upregulated by overexpression of ATF3 in glioblastoma cells. Furthermore, *klf6* is not upregulated in cells exposed to NO. We therefore suggest other pathways to be involved in the regulation of KLF6, although Xu *et al.*^53^ hypothesize a direct relation between ATF3 and KLF6. To investigate the involvement of ATF3 in the observed alterations after NO exposure, we analyzed gene expression after JS-K treatment in ATF3 overexpressing cells. The data showed that only *nfκb1* expression is further affected by NO what indicates that various pathways are involved besides the ATF3 signaling. No further upregulation in gene expression by NO in ATF3 overexpressing cells points out that ATf3 signaling is the major pathway triggered by NO. Many groups found STAT3 to be constitutively phosphorylated and activated in tumor cells, and inhibitors are already applied in tumor-immunology in patients.^54–56^ The phosphorylation status of STAT3 can give an indication of the malignancy and the proliferation of tumor cells. Downregulation of STAT3 and inhibition of phosphorylation is supposed to reduce migration and invasion capacity in glioma cells.^56,57^ In our study, we found gene expression of *stat3* upregulated by ATF3. Gene expression was not influenced by NO in ATF3 overexpressing cells. Even though *stat3* is upregulated by ATF3 it is no longer phosphorylated in ATF3 overexpressing cells as shown by Western blot. Only active STAT3 can interact with NF*κ*B and induce MMP expression.^54,58^ These data indicate that ATF3 can directly elicit MMP expression and activation. Furthermore, it recruits well-investigated signaling pathways to induce MMP expression and reduce migration and invasion in glioblastoma cells. Most of the MMPs are not detectable in healthy brain tissue and the presence of MMPs suggest a direct correlation to the malignancy of the tumor and the clinical outcome of the patient.^8,9,59^ In the past decade all clinical studies for MMP inhibition and immunotherapy failed and showed no benefit on migration and tumor progression.^60^ Therefore we conclude that ATF3 may be a novel target of migration in glioblastoma and should be further investigated.

## Materials and Methods

### Cell culture

The immortalized human glioma cell line U87MG obtained from ATCC (Manassas, VA, USA) and the primary glioblastoma cell line IC were cultured in Dulbecco’s modified Eagle medium (DMEM) containing 10% fetal bovine serum and 1% penicillin/streptavidin at 37 °C in a humidified atmosphere containing 5% CO_2_. The primary cell line was established from a surgical specimen of a patient (IC) with glioblastoma multiforme. Retrieval and scientific analysis of patient-derived tissue was approved by the local ethics committee under protocol 100020/09. The NO donor JS-K [*O*2-(2,4-dinitrophenyl)1 [(4-ethoxycarbonyl)piperazin-1-yl]diazen-1-ium-1,2-diolate] was synthesized as described earlier.^61^ Cells were exposed to JS-K concentrations between 0.5 and 25 *μ*M (stock solution 5.2 mM in DMSO) for up to 96 h when they reached 70–80% confluence. The final concentration of DMSO was not higher than 0.05% (when using 25 *μ*M JS-K).

### RNA interference and lentiviral transduction

Human shRNAs on lentiviral pGIPZ plasmid targeting ATF3 (V2LHS_172635, V3LHS_405369, V3LHS_352238, V3LHS_405368, V3LHS_352240, V3LHS_405370), overexpression of human ATF3 on lentiviral Precision LentiORF Collection plasmids (pLOC, pLOHS_100010432, pLOHS_100005353)) and negative controls (pGIPZ, pLOC) were transfected into HEK293T cells by cotransfection of lentiviral plasmids pCMV-MD2G and psPAX2 using HiPerFect transfection reagent (Qiagen, Hilden, Germany). Virus containing supernatant was collected and used for infection of U87 cells. shATF3 pLOHS_100010432 was found to knockdown ATF3 expression most efficiently and was used for the experiments as well as V3LHS_405370 for overexpression of ATF3. The functionality of the shRNAs and overexpression plasmids were validated by qRT-PCR analysis. Transduced cells were selected for puromycin resistance before further analysis.

### Migration assay

Cells were seeded in 24-well plates with silicon bars to create a gap of about 2 mm. Non-infected cells were treated with JS-K up to 3 *μ*M while cells overexpressing ATF3 or knockdown cells were not treated. After removing the bars cells started migrating from the edge of the wound and repopulated the gap area. The time required for wound closure was measured by microscopy (Zeiss Axiovert, Oberkochen, Germany) and documented up to 120 h.

### Invasion assay

Boyden chamber assay (BioCoat Matrigel, Corning Inc, NY, USA) was performed to assess the invasion ability of cells overexpressing ATF3 and knockdown. 2×10^4^ cells in serum-free medium were seeded per Boyden chamber and attracted with 10% FBS and 0.02 ng/ml PDGF. Cells were incubated for 21 h at 37 °C in a humidified atmosphere. Invaded cells were fixed in 4% PFA before the surface of matrigels was swabbed to remove non-invading cells. Crystal violet staining (0.2%) was performed according to manufacturer’s protocol. Cells were visualized by microscopy and quantified with ImageJ (National Institutes of Health (NIH), Bethesda, MD, USA; ×10, Zeiss Axiovert). Scale bars represent 500 *μ*m, frames show ×2.5 magnification.

### MTT-assay

Cell viability was determined by MTT Assay. Cells (10^4^) were grown in 96-well plates with complete DMEM and treated with 1–25 *μ*M JS-K for up to 96 h. MTT Assay was performed as described before.^28^ Absorbance at 570 nm was measured with Tecan Infinite200 (Tecan, Männedorf, Switzerland). Percentages were calculated relative to the viability of untreated controls set to 100%.

### Cell proliferation assay

Cell proliferation was monitored by 5′-bromodeoxyuridine (BrdU) incorporation assay (Roche Diagnostics, Mannheim, Germany). Three thousand cells were seeded in 96-well plates and treated with JS-K up to 3 *μ*M. Cells overexpressing ATF3 or knockdowns were not treated. Cells were stained with BrdU following the manufacturer’s instructions. The percentage of cells exhibiting genomic BrdU incorporation was measured by absorbance at 370 nm with Tecan Infinite200 (Tecan, Männedorf, Switzerland). Percentages were calculated relative to the proliferation of untreated controls or T0.

### Microarray

Gene expression changes were investigated by Microarray using the Illumina HumanHT-12 Expression BeadChip (Illumina Inc., San Diego, CA, USA) for analysis of 31 000 genes. The array was performed at the German Cancer Research Center (DKFZ), Heidelberg, Germany. RNA samples were purified from U87 treated with 15 *μ*M JS-K for 48 h as well as from untreated controls. Gene up- and downregulation caused by JS-K was evaluated. Microarray was done in triplicate.

### RNA purification, qRT-PCR and semiquantitative PCR

Total RNA was prepared from U87 and IC cells using the RNeasy mini kit according to the manufacturer’s instructions (Qiagen, Hilden, Germany). cDNA was generated from 1 *μ*g of total RNA in a volume of 30 *μ*l using M-MuLV reverse transcriptase (Thermo Fisher Scientific, Waltham, MA, USA) and 100 pmol of hexameric primers. cDNA was quantified by quantitative real-time PCR on a StepOnePlus System (Thermo Fisher Scientific) using SYBR™ Green Master Mix (Thermo Fisher Scientific) and specific primers for ATF3 (5′- CCTCTGCGCTGGAATCAGTC-3′ forward; 5′- TTCTTTCTCGTCGCCTCTTTTT-3′ reverse), TP53 (5′- AGGGCTCACTCCAGCCACCTG-3′ forward; 5′- AGAATGTCAGTCTGAGTCAGGCCCT-3′ reverse), NF*κ*B1 (5′- AACAGAGAGGATTTCGTTTCCG-3′ forward; 5′- TTTGACCTGAGGGTAAGACTTCT-3′ reverse), STAT3 (5′- CAGGAGGGCAGTTTGAGTCCCTCAC-3′ forward; 5′- GTCGTATCTTTCTGCAGCTTCCGTTCTC-3′ reverse), p38*α* (5′- CGAGCGTTACCAGAACCTGT-3′ forward; 5′- TGGAGAGCTTCTTCACTGCC-3′ reverse), KLF6 (5′- GGCAACAGACCTGCCTAGAG-3′ forward; 5′- AGGATTCGCTGACATCT-3′ reverse) and RPS18 (5′- TTTTGCGAGTACTCAACACCA-3′ forward; 5′- CCACACCCCTTAATGGCA-3′ reverse) as endogenous control. The conditions were 95 °C for 20 s, followed by 40 cycles of 3 s at 95 °C, 30 s at 60 °C. The relative expression level of the target gene compared with that of the housekeeping gene RPS18 was calculated with the 2^−ΔΔCt^ method and normalized to untreated control set to 1. The semiquantitative PCR was performed with Taq polymerase and buffers provided by Thermo Fisher Scientific. Specific primers were used for MMP1 (5′- GAGCTCAACTTCCGGGTAGA-3′ forward; 5′- CCCAAAAGCGTGTGACAGTA-3′ reverse), MMP2 (5′- GATACCCCTTTGACGGTAAGGA-3′ forward; 5′- CCTTCTCCCAAGGTCCATAGC-3′ reverse), MMP3 (5′- TGAAAGAGACCCAGGGAGTG-3′ forward; 5′- AGGGATTAATGGAGATGCCC-3′), MMP7 (5′- GGCCAAAGAATTTTTGCATC-3′ forward; 5′- GAGCTACAGTGGGAACAGGC-3′ reverse), MMP9 (5′- GCACTGCAGGATGTCATAGG-3′ forward; 5′- ACGACGTCTTCCAGTACCGA-3′ reverse), TIMP1 (5′- AGAGTGTCTGCGGATACTTCC-3′ forward; 5′- CCAACAGTGTAGGTCTTGGTG-3′ reverse), TIMP2 (5′- AAGCGGTCAGTGAGAAGGAAG-3′ forward; 5′- GGGGCCGTGTAGATAAACTCTAT-3′ reverse), TIMP3 (5′- GGTGAAGCCTCGGTACATCT-3′ forward; 5′- AGGACGCCTTCTGCAACTC-3′ reverse), TIMP4 (5′- GGCTCGATGTAGTTGCACAG-3′ forward; 5′- ACGCCTTTTGACTCTTCCCT-3′ reverse) and GAPDH (5′- GGCCTCCAAGGAGTAAGACC-3‘ forward; 5′- AGGGGTCTACATGGCAACTG-3‘ reverse) as endogenous control. A first cycle of 3 min at 95 °C was followed by 30 s at 95 °C, 30 s at 56 °C and 40 s at 72 °C for 40 cycles and finished with 72 °C for 10 min.

### Western blotting

U87 and primary IC glioma cells were cultured in DMEM containing 0.4% FBS. Supernatant of JS-K treated cells was collected and frozen at −20 °C. Cells for whole cell lysates were cultured in 10% FBS. Equal amounts of protein (4 *μ*g of supernatant, 20 *μ*g of lysate) were applied on 10% SDS-polyacrylamide gels and electrophoresed (BioRad, Munich, Germany). Proteins were blotted on PVDF-membranes by wet blotting (BioRad, Munich, Germany). Epitopes were blocked with 5% non-fat milk in tris-buffered saline with 0.05% Tween20 for 1 h at room temperature (RT). Blots were incubated with primary antibodies anti-ATF3 (ab87213 1 : 1000 Abcam, Cambridge, UK), anti-MMP2 (#4022 1 : 1000 Cell Signaling Technology, Inc., Danvers, MA, USA), anti-MMP7 (#MAB9071 1 : 1000 R&D System, Inc., Minneapolis, USA), anti-MMP9 (#3852 1 : 1000 Cell Signaling Technology, Inc.) anti-TIMP3 (#D74B10 1 : 1000 Cell Signaling Technology, Inc.), anti-GAPDH (1 : 10000 Abcam) overnight at 4 °C. After incubation with secondary antibodies goat anti-rabbit/mouse (Santa Cruz Biotechnology, Santa Cruz, CA, USA) for 1 h at RT proteins were visualized by enhanced chemiluminescence (BioRad). For loading control, a Coomassie-stained SDS-polyacrylamide gel was used for whole protein in the supernatant and GAPDH was used for the whole cell lysate.

### Gelatin zymography

Gelatin is a substrate of MMP2 and can be used for detection of activity in supernatant. Equal amounts of supernatant (4 *μ*g) used for Western blot were applied under non-reducing conditions on 10% copolymerized gelatin-polyacrylamide gels and electrophoresed (BioRad) in running buffer (25 mM Tris, 192 mM glycine, 0.1% SDS). Gels were washed 2 times in 2.5% Triton-X-100/ddH_2_O for 15 min following incubation in development buffer (50 mM Tris, 5 mM CaCl_2_, 0.02% NaN_3_) for 4 h. Gels were fixed by shaking in methanol:ethanol:acetic-acid (4.5 : 4.5 : 1) for 15 min and stained with fixation buffer containing 0.1% Coomassie for 2 h. Since gelatin is a protein, the whole gel is stained by Coomassie. Gels were incubated in fixation buffer until transparent bands appeared. The more gelatin is cleaved by MMP2, the more non-stained area is visible. Gels were visualized by a standard desktop scanner. Coomassie staining of total protein in conditioned media was used to demonstrate that equal numbers of cells were used during the conditioning of the media.

### Nuclear translocation of NF*κ*B

For nuclear translocation of NF*κ*B, 10 000 cells were cultured on 12 mm coverslips in complete medium. Cells were treated with 5 *μ*M JS-K for 48 h or 10 ng/ml TNF*α* for 6 h. Untreated cells were used as controls. Cells were fixed for 10 min with 4% PFA at RT and permeabilized for 15 min in 0.5% PBST at 4 °C. Coverslips were incubated with 2% bovine serum albumin/5% normal goat serum/PBS blocking solution for 30 min at 37 °C and 30 min at RT before 2 h incubation of *α* NF*κ*B p65 antibody (1 : 200, sc-8008 Santa Cruz, CA, USA) at RT in blocking solution. After washing with 0.05% PBST, cells were incubated with secondary goat *α* mouse antibody (Alexa 568, 1 : 5000, Invitrogen, Thermo Fisher, Bonn Germany) for 1 h at RT. Nuclei were counterstained with DAPI (10 ng/ml, Sigma-Aldrich, Steinheim, Germany) for 5 min and mounted with FluoromountG (Dako, Hamburg, Germany). Fluorescence was visualized by microscopy (×20, Zeiss Axio Observer). Scale bars represent 200 *μ*m.

### Statistical analysis

All experiments were performed in triplicates. Data are shown as mean±S.D. Data were compared using an unpaired two-tailed student’s *t*-test; *P*<0.05 was considered statistically significant.

## Figures and Tables

**Table 1:** Microarray analysis revealed genes upregulated by 15 *μ*M JS-K for 48 h

*Gene symbol*	*Gene function*	*Array fold change*	P-*value*
*HSPA6*	Interacting selectively with ATP, adenosine 5′-triphosphate, a universally important coenzyme and enzyme regulator	9.62	1.6449E−08
*CRYAB*	The action of a molecule that contributes to the structural integrity of the lens of an eye	4.83	2.5178E−08
*SERPINB2*	Stops, prevents or reduces the activity of serine-type endopeptidases, enzymes that catalyze the hydrolysis of nonterminal peptide bonds in a polypeptide chain	4.47	2.5761E−14
*HSPA7*	Interacting selectively with ATP, adenosine 5′-triphosphate, a universally important coenzyme and enzyme regulator	4.28	4.7973E−10
*RASD1*	Catalysis of the reaction: GTP+H_2_O=GDP+phosphate. Interacting selectively with GTP, guanosine triphosphate	2.75	6.3161E−08
*ANKRD1*	Transcription cofactor that represses transcription from a RNA polymerase II promoter; does not bind DNA itself. Interacting selectively with any protein or protein complex	2.59	1.547E−07
*ATF3*	The function of binding to a specific DNA sequence in order to modulate transcription. The transcription factor may or may not also interact selectively with a protein or macromolecular complex. The formation of a protein dimer, a macromolecular structure consists of two noncovalently associated identical or nonidentical subunits	2.51	4.1297E−19
*RRAD*	Catalysis of the reaction: GTP+H_2_O=GDP+phosphate. Interacting selectively with calmodulin, a calcium-binding protein	2.48	3.4441E−14
*GADD45B*	Interacting selectively with any protein or protein complex	2.08	1.8057E−10
*MMP10*	Catalysis of the hydrolysis of internal, alpha-peptide bonds in a polypeptide chain. Interacting selectively with calcium ions (Ca^2+^)	2.01	3.4869E−09

Fold changes are shown up to twofold. Microarray was done in triplicate, *P*-value indicated significance <0.05.
